# State-level prevalence, health service use, and spending vary widely among Medicare beneficiaries with Parkinson disease

**DOI:** 10.1038/s41531-019-0074-8

**Published:** 2019-01-24

**Authors:** Sneha Mantri, Michelle E. Fullard, James Beck, Allison W. Willis

**Affiliations:** 1 Parkinsons Disease Research, Education, and Clinical Center (PADRECC), Philadelphia VA Medical Center, 3900 Woodland Avenue, Philadelphia, PA 19104 USA; 2 Department of Neurology, University of Pennsylvania Perelman School of Medicine, Philadelphia, PA USA; 3 The Parkinson’s Foundation, New York, NY USA; 4 Department of Biostatistics, Epidemiology and Informatics, University of Pennsylvania, Philadelphia, PA USA; 5 Center for Clinical Epidemiology and Biostatistics, University of Pennsylvania Perelman School of Medicine, Philadelphia, PA USA

## Abstract

State-level variations in disease, healthcare utilization, and spending influence healthcare planning at federal and state levels and should be examined to understand national disparities in health outcomes. This descriptive study examined state-level variations in Parkinson disease (PD) prevalence, patient characteristics, Medicare spending, out-of-pocket costs, and health service utilization using data on 27.5 million Medicare beneficiaries in the US in 2014. We found that 45.8% (*n* = 179,496) of Medicare beneficiaries diagnosed with PD were women; 26.1% (*n* = 102,205) were aged 85+. The District of Columbia, New York, Illinois, Connecticut, and Florida had the highest age-, race-, and sex-adjusted prevalence of Parkinson disease among Medicare beneficiaries in the US. Women comprised over 48.5% of PD patient populations in West Virginia, Kentucky, Mississippi, Louisiana, and Arkansas. More than 31% of the PD populations in Connecticut, Pennsylvania, Hawaii, and Rhode Island were aged 85+. PD patients who were “dual-eligible”—receiving both Medicare and Medicaid benefits—also varied by state, from <10% to >25%. Hospitalizations varied from 304 to 653 stays per 1000 PD patients and accounted for 26.5% of the 7.9 billion United States Dollars (USD) paid by the Medicare program for healthcare services delivered to our sample. A diagnosis of PD was associated with greater healthcare use and spending. This study provides initial evidence of substantial geographic variation in PD patient characteristics, health service use, and spending. Further study is necessary to inform the development of state- and federal-level health policies that are cost-efficient and support desired outcomes for PD patients.

## Healthcare provision: Geographical variation across the United States

Analyses of the prevalence of Parkinson’s disease (PD), health service spending and use reveal significant disparities between different American states. Michelle Fullard at the University of Pennsylvania and colleagues examined data from 27.5 million over-65-year olds covered by national health insurance in the USA (Medicare). In 2014, Medicare spent nearly 7.9 billion USD on health care services for patients with PD, but the states with highest PD prevalence (District of Columbia, New York, and Illinois) did not always correlate with those registering the highest number of hospitalizations (New York, Michigan, and Illinois) or highest spending on healthcare provision for PD patients (Nevada, Texas, and Massachusetts). This type of study is becoming increasingly important to inform healthcare planning in countries with aging populations in which the prevalence of neurodegenerative diseases such as PD is steadily rising.

## Introduction

State-level variation in disease prevalence,^1,2^ health care utilization, spending/costs,^3,4^ healthcare quality,^5^ and clinical outcomes^6–10^ have been observed among Medicare beneficiaries. These data have driven health care reform initiatives and influenced health care planning at federal and state levels, in attempts to normalize spending and reduce inequity in care and outcomes. In the US, health care and reimbursement are increasingly governed at the state level. For instance, Medicare^11^ is a federally administered program providing health insurance to individuals over the age of 65, while Medicaid, which provides coverage to individuals below the poverty line, is funded by individual states.^11^ Persons covered by both programs are termed “dual-eligibles”. Medicare beneficiaries who are dual-eligible and have a neurodegenerative disease, like Parkinson disease (PD), have often qualified for Medicaid due to loss of wealth from health care expenses and/or long-term care services. For dual-eligible individuals over the age of 65, Medicare remains the primary payer for office visits, hospitalizations, home health, and skilled nursing facility care; Medicaid assists with remaining costs of care.

PD is a common neurodegenerative condition marked by sociodemographic disparities in care and outcomes.^12–14^ However, there are limited population-level data on geographic variations in PD, and no data on how PD care and spending differ across the US. With the increasing prevalence of PD in the US, health care needs and costs will also increase, so population-level data is needed to inform health policy and planning at the state and federal level to address these changing needs. To address these gaps in knowledge, this descriptive study examined state-level variation in PD prevalence among US Medicare beneficiaries. We also examined state-level variations in PD patient characteristics, Medicare spending, out-of-pocket health care costs, and health service utilization. These data are useful for targeting areas in which PD patients may have increased need and can be used to evaluate the effects of future changes in Medicare and Medicaid policies on persons with PD.

## Results

### Variation in PD prevalence and characteristics

**Fig. 1:**
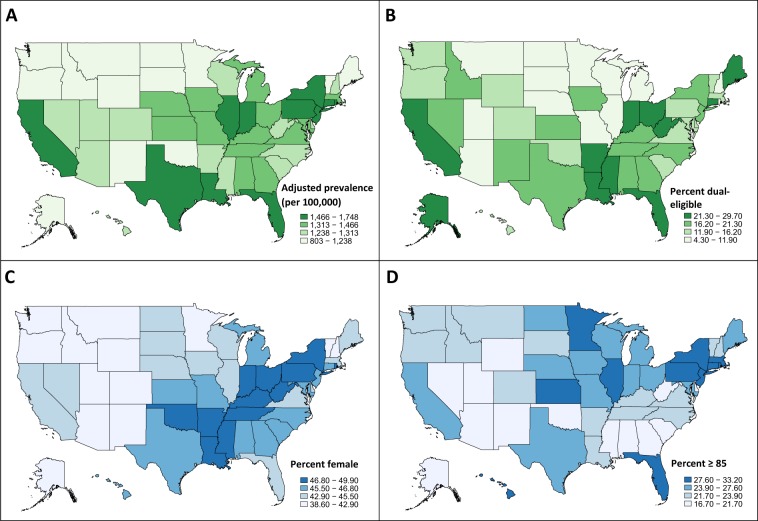
Prevalence of Parkinson’s disease and characteristics of individuals with PD by state. **a** Prevalence of Parkinson’s disease (per 100,000), adjusted for age, race, and sex, among Medicare beneficiaries in 2014. **b** Percentage of Medicare PD population that is dual-eligible. **c** Percentage of Medicare PD population that is female. **d** Percentage of Medicare PD population aged 85 years and older. Data are shown in quartiles

We identified 27,538,023 Medicare beneficiaries that met our inclusion criteria, of whom 392,214 had a PD diagnosis in 2014. State-level variation in the prevalence of PD per 100,000 Medicare individuals is shown in Table 1 and Fig. 1. Crude prevalence varied from 845/100,000 in Minnesota to 1781/100,000 in New York. The top five states—New York, Connecticut, Florida, Pennsylvania, and Rhode Island—contained 20.7% of all Medicare beneficiaries diagnosed with PD in our sample. After adjusting for baseline differences in race, age, and sex, New York, Illinois, Connecticut, Florida, Pennsylvania, and Rhode Island remained the states with the highest prevalence.

**Table 1:** PD prevalence and demographic characteristics among Medicare beneficiaries, by state

	Medicare beneficiaries total, *n*	PD, *n*	Crude prevalence per 100,000 (95% CI)	Age, race, sex adjusted PD prevalence (per 100,000)^a^	Adjusted PD prevalence rank	% Female	Rank % female	% 65–69	% 70–74	% 75–79	% 80–84	% 85+	Rank % 85+	% Dual eligible	Rank % dual eligible
Alabama	512,181	7138	1393.64 (1361.81, 1426.02)	1459.18	14	46.7%	14	13.4%	19.8%	23.9%	21.9%	21.0%	45	18.6%	19
Alaska	59,735	604	1011.13 (933.25, 1093.75)	1086.54	47	38.6%	51	17.7%	24.8%	23.3%	17.4%	16.7%	51	22.7%	10
Arizona	529,547	6676	1260.70 (1230.91, 1291.01)	1279.80	33	39.7%	48	14.5%	20.3%	23.1%	20.7%	21.3%	43	9.2%	48
Arkansas	343,037	4415	1287.03 (1249.72, 1325.16)	1288.81	32	48.6%	5	12.5%	19.7%	23.4%	22.3%	22.0%	37	23.5%	7
California	2,248,672	34,101	1516.49 (1500.58, 1532.40)	1520.10	10	44.9%	29	12.2%	17.6%	20.9%	21.7%	27.6%	12	28.7%	2
Colorado	372,747	4524	1213.69 (1178.91, 1249.22)	1237.98	38	40.9%	47	15.1%	19.5%	21.1%	20.5%	23.8%	28	15.5%	29
Connecticut	350,160	5904	1686.08 (1643.84, 1729.13)	1560.43	4	46.2%	17	10.3%	15.3%	19.0%	22.3%	33.2%	1	25.7%	5
Delaware	126,971	1560	1228.62 (1169.14, 1290.33)	1272.21	36	43.1%	38	14.7%	19.9%	22.7%	21.5%	21.3%	42	15.3%	31
District of Columbia	52,694	646	1225.94 (1134.64, 1322.60)	1747.75	1	45.5%	25	12.4%	17.3%	22.6%	21.5%	26.2%	20	29.7%	1
Florida	1,875,551	30,653	1634.34 (1616.27, 1652.41)	1551.08	5	44.8%	31	10.5%	16.8%	21.8%	22.7%	28.2%	9	23.3%	9
Georgia	745,589	9251	1240.76 (1215.82, 1265.96)	1322.34	25	45.7%	23	13.1%	20.1%	23.6%	21.9%	21.3%	41	18.9%	18
Hawaii	89,335	1114	1246.99 (1175.79, 1321.34)	1265.80	37	45.6%	24	11.6%	16.3%	19.1%	21.7%	31.2%	3	12.0%	37
Idaho	141,265	1594	1128.37 (1074.29, 1184.46)	1129.67	43	42.3%	42	11.7%	19.1%	24.7%	22.2%	22.3%	35	16.8%	24
Illinois	1,236,916	19,466	1573.75 (1551.93, 1595.63)	1566.12	3	45.5%	26	11.5%	16.8%	21.4%	22.3%	28.0%	10	11.2%	41
Indiana	655,313	9751	1487.99 (1458.89, 1517.34)	1472.26	12	47.6%	9	12.7%	17.3%	22.5%	22.1%	25.4%	22	24.3%	6
Iowa	395,093	5504	1393.08 (1356.89, 1429.82)	1323.44	24	44.7%	32	12.4%	17.3%	22.2%	22.3%	25.8%	21	17.6%	20
Kansas	323,255	4977	1539.65 (1497.64, 1582.53)	1455.60	15	46.1%	18	12.2%	16.1%	20.9%	22.1%	28.7%	8	17.5%	21
Kentucky	432,696	6154	1422.24 (1387.28, 1457.85)	1432.57	18	49.3%	2	13.6%	18.3%	23.1%	21.8%	23.2%	31	20.1%	16
Louisiana	381,276	5466	1433.60 (1396.24, 1471.70)	1518.47	11	48.9%	4	13.4%	18.8%	23.1%	22.9%	21.8%	38	21.9%	12
Maine	168,276	2187	1299.65 (1246.37, 1354.60)	1237.83	39	44.4%	34	11.9%	17.4%	20.4%	22.7%	27.6%	13	26.0%	4
Maryland	651,007	8612	1322.87 (1295.33, 1350.84)	1414.82	20	46.5%	15	12.6%	18.0%	22.0%	21.1%	26.3%	19	15.5%	30
Massachusetts	675,050	9879	1463.44 (1435.00, 1492.13)	1420.75	19	45.0%	28	12.3%	17.4%	20.2%	21.4%	28.8%	7	15.9%	27
Michigan	928,374	13,097	1410.74 (1386.90, 1434.74)	1397.62	22	45.9%	20	12.0%	17.7%	21.0%	21.9%	27.4%	15	11.9%	39
Minnesota	548,458	4635	845.09 (821.12, 869.56)	802.88	51	42.1%	43	10.6%	16.4%	20.5%	24.7%	27.7%	11	5.2%	50
Mississippi	337,839	4130	1222.47 (1185.83, 1259.94)	1312.87	26	49.1%	3	13.7%	19.9%	24.3%	22.7%	19.4%	49	27.3%	3
Missouri	593,862	8745	1472.56 (1442.16, 1503.43)	1443.32	16	46.5%	16	12.9%	18.5%	21.7%	21.9%	25.0%	23	11.0%	42
Montana	127,884	1328	1038.44 (983.98, 1095.11)	1089.36	46	38.9%	50	15.3%	19.3%	22.1%	21.1%	22.3%	36	10.8%	43
Nebraska	217,187	3214	1479.83 (1429.69, 1531.26)	1403.70	21	43.7%	36	11.6%	16.7%	21.8%	22.4%	27.5%	14	10.2%	45
Nevada	214,117	2598	1213.35 (1167.63, 1260.39)	1306.13	28	43.4%	37	15.7%	22.0%	24.0%	19.5%	18.8%	50	17.3%	22
New Hampshire	180,003	2405	1336.08 (1283.82, 1389.91)	1297.98	30	41.7%	44	13.6%	18.9%	21.9%	21.7%	23.9%	26	6.1%	49
New Jersey	946,172	14,871	1571.70 (1546.78, 1596.91)	1541.23	8	45.9%	21	11.2%	17.1%	20.6%	22.2%	28.9%	6	17.0%	23
New Mexico	175,502	1805	1028.47 (982.08, 1076.49)	1010.21	49	42.8%	40	14.0%	19.0%	24.8%	22.3%	19.9%	48	20.4%	15
New York	1,468,850	26,160	1780.98 (1759.69, 1802.27)	1719.80	2	47.5%	10	11.2%	15.7%	20.4%	22.2%	30.5%	5	19.7%	17
North Carolina	907,156	11,008	1213.46 (1191.08, 1236.04)	1291.97	31	45.8%	22	13.8%	19.5%	22.5%	21.7%	22.6%	34	21.3%	13
North Dakota	93,148	1180	1266.80 (1196.48, 1340.14)	1122.78	44	44.9%	30	11.1%	16.4%	21.6%	24.0%	26.9%	16	4.3%	51
Ohio	928,421	13,485	1452.46 (1428.28, 1476.80)	1439.76	17	47.1%	11	13.0%	17.5%	21.6%	21.3%	26.6%	18	23.4%	8
Oklahoma	413,847	5045	1219.04 (1185.95, 1252.82)	1222.55	40	47.0%	12	13.7%	18.6%	24.0%	22.0%	21.7%	39	13.9%	33
Oregon	299,606	3331	1111.79 (1074.71, 1149.81)	1151.28	42	41.2%	46	15.0%	21.0%	22.4%	18.6%	23.1%	32	12.0%	38
Pennsylvania	1,080,256	17,272	1598.88 (1575.35, 1622.66)	1549.18	6	48.2%	7	11.6%	15.5%	20.1%	21.4%	31.2%	2	12.7%	35
Rhode Island	81,781	1288	1574.93 (1491.31, 1661.99)	1543.40	7	48.2%	8	13.4%	15.1%	19.3%	21.0%	31.1%	4	11.5%	40
South Carolina	540,745	6371	1178.18 (1149.69, 1207.20)	1274.95	35	46.0%	19	15.2%	20.8%	22.4%	20.9%	20.6%	46	13.1%	34
South Dakota	116,221	1352	1163.30 (1102.86, 1226.17)	1053.87	48	44.0%	35	13.4%	17.4%	23.4%	22.1%	23.7%	30	10.3%	44
Tennessee	597,675	8595	1438.07 (1408.12, 1468.32)	1466.00	13	48.4%	6	13.7%	19.2%	22.2%	22.1%	22.9%	33	16.3%	25
Texas	1,859,155	28,075	1510.09 (1492.63, 1527.57)	1521.95	9	46.8%	13	12.8%	18.7%	22.3%	22.1%	24.1%	24	20.5%	14
Utah	165,047	2232	1352.34 (1297.46, 1408.92)	1299.46	29	39.2%	49	12.7%	20.3%	24.6%	22.3%	20.1%	47	9.8%	46
Vermont	90,310	1035	1146.05 (1078.18, 1217.04)	1120.26	45	42.9%	39	12.5%	18.7%	23.0%	22.0%	23.8%	27	16.2%	26
Virginia	838,962	10,990	1309.95 (1285.78, 1334.32)	1366.83	23	44.6%	33	12.8%	19.1%	22.0%	22.4%	23.7%	29	12.7%	36
Washington	599,741	7424	1237.86 (1210.11, 1266.08)	1215.55	41	42.5%	41	12.4%	19.6%	22.6%	21.4%	23.9%	25	15.9%	28
West Virginia	216,202	2745	1269.64 (1223.10, 1317.49)	1307.36	27	49.9%	1	15.3%	19.6%	23.9%	20.0%	21.1%	44	22.2%	11
Wisconsin	532,630	6960	1306.72 (1276.48, 1337.48)	1279.27	34	45.5%	27	13.0%	16.6%	21.5%	22.2%	26.8%	17	9.6%	47
Wyoming	72,506	662	913.02 (845.73, 984.23)	883.63	50	41.5%	45	14.4%	21.8%	21.1%	21.3%	21.5%	40	14.2%	32

^a^Age-, race-, and sex-adjusted prevalence of PD using the direct method of standardization. Standard population was total Medicare beneficiary population

State-level estimates of PD prevalence among adults aged 45 years and older were considerably lower, ranging from 450/100,000 in Alaska to 668/100,000 in Florida. Prevalence estimates between the two samples were most similar in Wyoming, New Mexico, Montana, Oregon, and Idaho, all states with very low prevalence in general. In states with the greatest number of Medicare beneficiaries (New York, Texas, Connecticut, Illinois, and New Jersey), Medicare prevalence estimates were more than 2.25 times greater than estimates which included younger individuals (e-Table 1).

Nationally, 45.8% (*n* = 179,496) of individuals diagnosed with PD in our Medicare dataset were women, and 26.1% (*n* = 102,205) were aged 85 and above. West Virginia, Kentucky, Mississippi, Louisiana, and Arkansas had the largest proportions of PD patients who were women, over 48.5% of their state PD populations. The proportion of PD patients over the age of 85 was greatest in Connecticut (33.2%), Pennsylvania (31.2%), Hawaii (31.2%), and Rhode Island (31.1%). In contrast, less than 19% of the PD populations in Alaska and Nevada were in the oldest age group. The percentage of individuals diagnosed with PD who were dual-eligible similarly varied by state, from <10% in North Dakota, Minnesota, New Hampshire, Arizona, Wisconsin, and Utah, to >25% in Connecticut, Maine, Mississippi, California, and the District of Columbia (Fig. 1).

### Health care utilization

In the year 2014, our Medicare PD sample had 219,049 hospitalizations (558 per 1000 PD); 37,839 readmissions (172 per 1000 hospitalizations); 3,699,767 outpatient physician office visits (9433 per 1000 PD); 34,159 hospice stays (87 per 1000 PD); 113,027 skilled nursing facility stays (288 per 1000 PD); 466,160 emergency room visits (1188 per 1000 PD), of which 39.0% resulted in hospital admission; 1,308,934 durable medical equipment events (3337 per 1000 PD); 6,676,119 laboratory tests (17,021 per 1000 PD); 2,435,654 imaging events (6210 per 1000 PD); and 4,879,538 home health visits (12,441 per 1000 PD). The portion of our sample that had prescription coverage had 16.5 million prescription events.

**Fig. 2:**
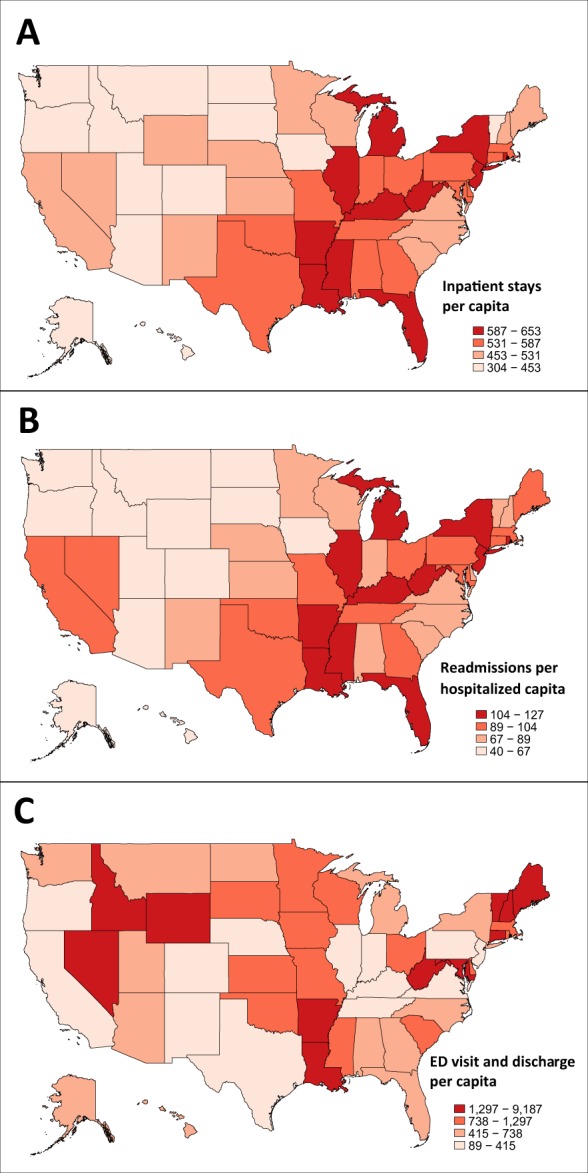
ER visits and hospitalizations in the PD population by state. **a** Number of acute inpatient stays, per 1000 PD patients, in Medicare beneficiaries with PD in 2014. **b** Number of readmissions, per 10,000 hospitalized PD patients, in Medicare beneficiaries with PD in 2014. **c** Number of ED visits with discharge, per 1000 PD patients, in Medicare beneficiaries with PD in 2014. Data are shown in quartiles

As shown in Table 2 and Fig. 2, Medicare beneficiaries with PD in Hawaii, Alaska, Utah, North Dakota, and Idaho had the lowest per capita number of hospitalizations (from 304 to 384 per 1000 PD). This was nearly half the hospitalization per capita rate found in New York, Michigan, Illinois, West Virginia, and Florida (624–653 hospital stays per 1000 PD). Thirty-day readmissions have become an increasingly used metric for performance evaluations and reimbursement guidelines. The readmission rate, which varied less by state, was highest in Florida (127 per 1000 hospitalizations), and greater than 115 per 1000 hospitalizations in the District of Columbia, New York, Michigan, and Arkansas. The lowest readmission rates per capita (less than 50 per 1000 hospitalizations) were found in Utah, North Dakota, South Dakota, Alaska, and Hawaii.

**Table 2:** State-level health care utilization among Medicare beneficiaries diagnosed with PD, 2014

State name	PD	Acute inpatient stays			Readmissions			Emergency department visit with admission			Emergency department visit without admission			Emergency department admission	
	*n*	*n*	Per 1000 PD patients	Rank	*n*	Per 10000 hospitalized PD patients	Rank	*n*	Per 1000 PD patients	Rank	*n*	Per 1000 PD patients	Rank	%	Rank
Alabama	7138	3996	560	20	626	88	28	3277	459	22	3646	511	35	47.3	15
Alaska	604	206	341	50	26	43	50	125	207	50	387	641	32	24.4	36
Arizona	6676	2952	442	41	448	67	39	2326	348	36	4299	644	31	35.1	25
Arkansas	4415	2730	618	7	513	116	5	2143	485	13	16,078	3642	4	11.8	48
California	34,101	17,818	523	29	3224	95	21	15,366	451	24	7347	215	45	67.7	6
Colorado	4524	1907	422	43	229	51	46	1480	327	37	801	177	49	64.9	8
Connecticut	5904	3358	569	17	582	99	18	3044	516	9	20,459	3465	5	13.0	45
Delaware	1560	835	535	25	120	77	32	722	463	19	2008	1287	15	26.4	32
District of Columbia	646	383	593	12	80	124	2	348	539	5	2735	4234	3	11.3	49
Florida	30,653	20,011	653	1	3885	127	1	17,913	584	1	20,166	658	29	47.0	17
Georgia	9251	5054	546	24	867	94	23	4195	453	23	4683	506	36	47.3	16
Hawaii	1114	339	304	51	45	40	51	307	276	46	729	654	30	29.6	28
Idaho	1594	612	384	47	84	53	45	383	240	48	2656	1666	11	12.6	47
Illinois	19,466	12,269	630	3	2139	110	8	10,160	522	7	3641	187	47	73.6	3
Indiana	9751	5418	556	21	869	89	26	4176	428	28	1822	187	48	69.6	4
Iowa	5504	2491	453	39	356	65	41	1601	291	42	4485	815	20	26.3	33
Kansas	4977	2618	526	28	378	76	33	1751	352	35	5295	1064	19	24.9	35
Kentucky	6154	3828	622	6	644	105	12	2987	485	14	546	89	51	84.5	1
Louisiana	5466	3296	603	10	596	109	9	2613	478	16	9194	1682	10	22.1	39
Maine	2187	1159	530	27	215	98	19	861	394	31	5511	2520	6	13.5	44
Maryland	8612	4838	562	19	866	101	15	4233	492	12	11,750	1364	12	26.5	31
Massachusetts	9879	5777	585	14	1026	104	14	5135	520	8	7376	747	24	41.0	19
Michigan	13,097	8250	630	4	1542	118	4	7108	543	4	9662	738	26	42.4	18
Minnesota	4635	2170	468	36	331	71	36	1511	326	38	3446	743	25	30.5	27
Mississippi	4130	2545	616	8	440	107	10	1938	469	17	4786	1159	17	28.8	29
Missouri	8745	5057	578	16	879	101	16	3861	442	26	6785	776	23	36.3	24
Montana	1328	591	445	40	87	66	40	366	276	45	921	694	28	28.4	30
Nebraska	3214	1498	466	37	225	70	38	878	273	47	1334	415	39	39.7	22
Nevada	2598	1380	531	26	261	100	17	1211	466	18	4451	1713	9	21.4	40
New Hampshire	2405	1186	493	32	201	84	29	922	383	32	22,095	9187	1	4.0	50
New Jersey	14,871	9014	606	9	1658	111	7	8235	554	2	3679	247	44	69.1	5
New Mexico	1805	840	465	38	133	74	35	636	352	34	553	306	40	53.5	11
New York	26,160	16,327	624	5	3129	120	3	14,291	546	3	14,039	537	34	50.4	13
North Carolina	11,008	5374	488	34	851	77	31	4509	410	29	4688	426	38	49.0	14
North Dakota	1180	446	378	48	52	44	48	222	188	51	844	715	27	20.8	41
Ohio	13,485	7910	587	13	1406	104	13	6519	483	15	10,690	793	21	37.9	23
Oklahoma	5045	2787	552	23	475	94	22	2250	446	25	6541	1297	13	25.6	34
Oregon	3331	1324	397	45	192	58	42	963	289	43	917	275	42	51.2	12
Pennsylvania	17,272	10,026	580	15	1685	98	20	8827	511	10	4950	287	41	64.1	9
Rhode Island	1288	773	600	11	135	105	11	678	526	6	1019	791	22	40.0	21
South Carolina	6371	3163	496	31	493	77	30	2565	403	30	8260	1296	14	23.7	37
South Dakota	1352	527	390	46	59	44	49	310	229	49	1471	1088	18	17.4	43
Tennessee	8595	4761	554	22	781	91	25	3959	461	21	970	113	50	80.3	2
Texas	28,075	15,779	562	18	2562	91	24	12,951	461	20	7552	269	43	63.2	10
Utah	2232	839	376	49	102	46	47	633	284	44	1231	552	33	34.0	26
Vermont	1035	441	426	42	73	71	37	304	294	41	2091	2020	7	12.7	46
Virginia	10,990	5648	514	30	969	88	27	4835	440	27	2333	212	46	67.5	7
Washington	7424	2957	398	44	423	57	43	2291	309	39	3317	447	37	40.9	20
West Virginia	2745	1789	652	2	314	114	6	1373	500	11	5515	2009	8	19.9	42
Wisconsin	6960	3430	493	33	526	76	34	2563	368	33	8878	1276	16	22.4	38
Wyoming	662	322	486	35	37	56	44	197	298	40	5476	8272	2	3.5	51

Approximately 7.9 billion United States Dollars (USD) were paid by the Medicare program for health care services delivered to our PD sample in 2014. The costliest services were inpatient care (2.1 billion USD), skilled nursing facility care (1.4 billion USD), hospital outpatient care (881.0 million USD), and home health (776.5 million USD). For all health care services, Medicare and out-of-pocket spending was significantly higher for beneficiaries with PD than for beneficiaries without PD (e-Table 2).

**Fig. 3:**
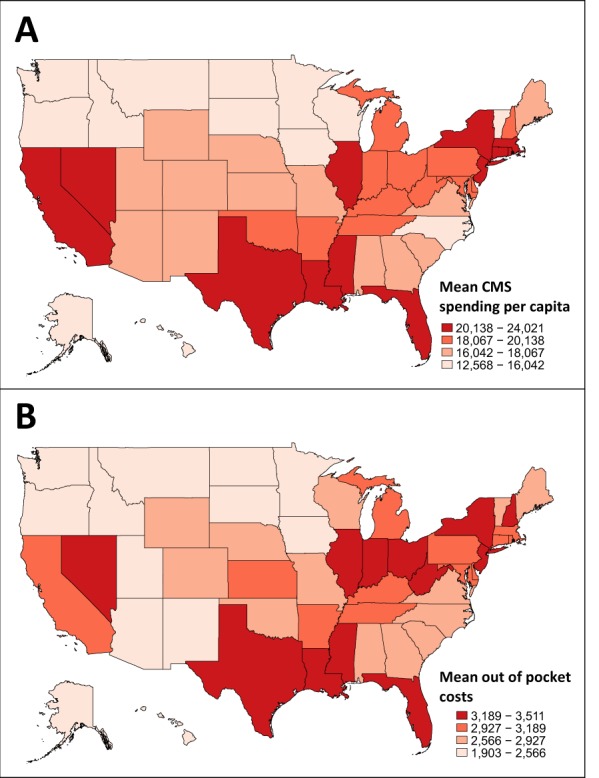
Medicare spending and out of pocket costs by state. **a** Mean CMS spending per capita for Medicare beneficiaries with PD, 2014. **b** Out of pocket costs per Medicare beneficiary with PD, 2014. Data are shown in quartiles

There was significant state and regional variation in per capita CMS and out of pocket costs (Fig. 3 and Table 3). The top five states for CMS spending were Nevada, Texas, Massachusetts, Florida, and New York (all greater than 22,000 USD per beneficiary with PD), almost double what was spent in South Dakota and Hawaii. Beneficiary responsibility is proportional to CMS spending; therefore, states in the top quartile for CMS spending were also in the top quartile for out-of-pocket costs. The highest out-of-pocket costs were in the Great Lakes, northeast, and south-central regions. The lowest costs were in the Pacific Northwest, mountain regions, and parts of the South.

**Table 3:** State-level Medicare and beneficiary out of pocket spending among Medicare beneficiaries diagnosed with PD, 2014

State name	Spending by payer in 2014 US Dollars					
	CMS			Beneficiary/out of pocket		
	Mean	Std dev.	Rank	Mean	Std dev.	Rank
Alabama	17,623	23,830	29	2705	3814	34
Alaska	12,954	22,376	49	2034	2745	50
Arizona	17,447	26,231	31	2433	3337	42
Arkansas	18,707	26,877	23	3025	4474	23
California	22,774	38,562	8	3071	4978	21
Colorado	16,722	25,346	35	2613	3944	36
Connecticut	22,002	31,726	9	3178	4523	14
Delaware	19,495	29,910	21	2956	4061	24
District of Columbia	19,703	36,094	20	3097	4674	19
Florida	23,193	29,918	4	3509	4724	2
Georgia	18,067	27,984	26	2873	4363	28
Hawaii	12,568	22,087	51	1903	2896	51
Idaho	14,839	21,867	44	2369	3420	47
Illinois	21,315	30,363	11	3458	4872	4
Indiana	19,867	28,425	15	3472	4913	3
Iowa	14,262	21,739	45	2566	3490	39
Kansas	17,775	24,855	27	2951	4052	25
Kentucky	18,576	26,232	24	3189	4524	13
Louisiana	22,805	31,423	7	3511	5223	1
Maine	17,657	25,555	28	2814	3650	30
Maryland	19,826	30,968	16	3079	4399	20
Massachusetts	23,323	33,708	3	3130	4570	17
Michigan	20,138	28,494	13	3125	4355	18
Minnesota	16,042	24,007	39	2415	3362	44
Mississippi	20,522	29,278	12	3295	5023	9
Missouri	17,588	24,837	30	2927	3939	26
Montana	14,261	22,294	46	2418	3136	43
Nebraska	16,221	24,359	38	2925	4010	27
Nevada	24,021	38,435	1	3202	5092	12
New Hampshire	20,000	30,846	14	3221	4782	10
New Jersey	22,884	33,858	6	3430	4881	5
New Mexico	17,010	24,964	34	2515	3916	40
New York	22,960	36,240	5	3358	4842	6
North Carolina	15,983	22,996	40	2742	3979	32
North Dakota	13,380	25,279	48	2409	3331	45
Ohio	19,765	28,555	19	3210	4594	11
Oklahoma	19,788	28,698	18	2746	4373	31
Oregon	13,696	21,428	47	2072	2859	49
Pennsylvania	19,816	28,253	17	3151	4368	16
Rhode Island	21,870	31,332	10	3154	4596	15
South Carolina	17,240	25,346	33	2715	4182	33
South Dakota	12,932	20,101	50	2407	3347	46
Tennessee	18,102	25,362	25	3049	4456	22
Texas	23,512	32,617	2	3340	5030	7
Utah	16,691	25,062	36	2326	3475	48
Vermont	15,855	25,053	42	2588	3536	38
Virginia	16,682	25,574	37	2691	3859	35
Washington	15,320	25,069	43	2437	3712	41
West Virginia	19,440	28,263	22	3299	4725	8
Wisconsin	15,919	26,427	41	2613	3768	37
Wyoming	17,407	26,752	32	2865	4116	29

## Discussion

In this descriptive study, we determined that among Medicare beneficiaries, there is significant state-level variation in PD prevalence, demography, dual-eligible status, and spending. States which have a higher prevalence of PD may have a larger proportion of high-risk factor patient groups, a higher concentration of providers who recognize and document PD, increased public awareness of PD symptoms, or increased health care seeking behaviors among people living in the state. Among our top PD prevalence states, Florida and New York also rank high in terms of absolute number of Medicare beneficiaries, and have large supplies of health care providers. Environmental factors, including exposure to exogenous toxicants (such as pesticides,^15^ heavy metals,^16^ or solvents^17^) vary by location and may influence our prevalence estimates by altering the risk of PD or of a PD diagnosis. There are proposed protective factors for PD, such as coffee consumption^18,19^ and exercise habits,^20,21^ but it is not clear whether these vary sufficiently across states to impact PD prevalence estimates. Finally, prevalence calculations can be impacted by differential mortality. Future research will seek to understand the geographic variation of PD in terms of differences in risk, mortality, and diagnostic accuracy.

The strongest risk factor for PD is age.^22,23^ Therefore, it is not surprising that PD prevalence estimates for individuals aged 45 and above were substantially lower than those estimated using a population sample aged 65 and older. These lower prevalence estimates reflect the uncommonness of PD diagnoses among individuals below the age of 60 and highlights the importance of presenting age-stratified data for PD burden estimates, particularly if that data includes very low-risk subpopulations.

The geographic variation in the proportion of dual-eligible individuals among PD is more challenging to explain. The most concerning potential contributing factors to high proportions of dual eligibles in a state are increased need for permanent nursing facility care due to suboptimal management of PD, or an increased incidence of outcomes that precipitate nursing home placement, such as cognitive impairment or falls with injury. Ease of obtaining Medicaid may also explain a portion of our findings; states with above-average percentages of dual eligible may have a higher relative income threshold for Medicaid eligibility, or formal/informal processes in place that facilitate Medicaid receipt. While Medicaid eligibility is administered at the state level, federal subsidies are given to states to offset the costs of the program. The amount of federal support varies from state to state, as decided by state leaders. For example, the District of Columbia, California, Arkansas, Ohio, and Connecticut, which had some of the highest proportions of dual eligible PD patients, had also opted to expand Medicaid eligibility as part of the Affordable Care Act (ACA), and had done so by 2014. ACA-supported Medicaid expansion was not designed to impact older dual eligibles; however, there may be spillover effects that result in the increased pursuit of Medicaid eligibility by PD patients in these states. Other states in the top quartile for dual eligibles—Mississippi, Louisiana, and Indiana—also have the highest proportions of individuals living at or below the poverty line.^24^ The interplay between the need for long-term care services, subsidies, income, and Medicaid eligibility is complex. Future studies will determine how PD patients may be uniquely impacted by state and federal level Medicare policies.

We noted in our sample that women comprised close to half of the PD population in some states. Other epidemiologic studies have shown that the incidence and prevalence of PD among women is lower than that of men.^25,26^ It is important to point out the distinction between disease prevalence and proportion of a disease population with a specific characteristic. When CMS datasets are used to calculate PD prevalence and incidence, the expected male:female ratio of 1.5:1 is observed.^27^ In this study, we focused our sex data calculations on the PD sample alone and report the proportion of Medicare beneficiaries diagnosed with PD that is female, *not* the prevalence of PD among female Medicare beneficiaries. Female Medicare beneficiaries outnumber male beneficiaries, and women have a greater life expectancy, both in the general population^28^ and among individuals with PD.^29^ Thus, our finding that nearly half of Medicare beneficiaries with PD are female is expected. Although women diagnosed with PD are a sizable portion of the PD population, they are highly under-represented in PD research and clinical trials. Recent data suggests that current payer models and care patterns do not meet the needs of women with PD, who have less access to specialized care and greater unreimbursed care needs.^13,30^ Improving PD outcomes will require increased attention to women with PD, from both research and clinical perspectives, especially given that almost half of the Medicare PD population is made up of women.

The concept of comparing Medicare utilization and cost by state was pioneered by the Dartmouth Atlas of Health Care, and their data showing significant variation has led to efforts to improve health systems across the US.^31–33^ In the general population, such variation is suggested to be due to regional differences in health care seeking behavior, increased need due to greater comorbid disease burden or social determinants of health, or increased availability of providers.^2,34^ Hospitalization for PD specialist care, such as deep brain stimulator (DBS) implantation, could contribute to our observed differences, particularly in states with multiple academic centers, however previous research has demonstrated that DBS use among Medicare beneficiaries diagnosed with PD is very low.^14^ In particular, our data on hospitalizations and readmissions do not follow a pattern consistent with provider availability. Excess hospitalizations and readmissions of PD patients occurred in Southern and Midwest states, which are known to have health provider shortages. Future studies will examine the nature of hospitalizations of PD patients and determine the extent to which they are PD related or avoidable (i.e., due to medication misadventure, ambulatory care sensitive condition).

Not surprisingly, we found that beneficiaries with PD have increased health care utilization and spending compared to those without PD, which is consistent with prior, smaller studies performed in the US.^35,36^ This was true across all sectors of care (inpatient, outpatient, skilled nursing, and ancillary services), and is in line with other data demonstrating that PD, its complications, and the shift away from comorbid disease care and prevention that occurs after a PD diagnosis drive health care spending and utilization among these individuals.^12,37,38^ On average, 20,142 Medicare dollars were spent per beneficiary with PD, with the lowest spent in Hawaii (12,568 USD per PD beneficiary) and the highest in Nevada (24,021 USD per PD beneficiary). Comparison of cost with other countries is difficult due to differing methodologies, inclusion of direct and indirect costs, and usually much smaller study populations, however, a comprehensive review on the subject has been done.^39^ PD costs in the US are most similar to Germany,^40^ the UK,^41^ and Australia^42^ and higher than those in Sweden, Finland, Austria, Italy, Portugal, Russia, and the Czech Republic.^43,44^ Hospitalizations were the main driver of cost in many of these studies.

By examining state-level variation in out-of-pocket and CMS payments, we identified regions of high and low spending, which are not consistently the regions with the highest PD prevalence. Variation in spending patterns may be due to local practice patterns,^7,45^ migration patterns of higher-risk individuals,^2,46^ or both. The proportion of state expenditures related to PD care will rise as PD prevalence increases; research to understand these variations is necessary to develop policies aimed at reducing state health care expenditures associated with undesired patient or clinical outcomes.^47^ In particular, economic burden data that includes the younger PD population is needed, not only to provide a complete picture of the economic burden of PD, but also because younger individuals with PD are less likely to have comorbid conditions. Thus, in this age group, medical expenditures may more directly reflect PD care costs alone.

Our study provides a comprehensive assessment of state-level variation in PD prevalence and spending patterns among the Medicare population. Nevertheless, some important limitations should be noted. We relied on administrative claims data from a single year, which may not be representative of broader secular trends in PD care. Medicare administrative claims data have been shown to be both accurate and valid^48^ and are commonly used in studies of spending, enabling comparison to other chronic diseases. Medicare data obtained for research purposes has been subject to a strict quality assurance process. Nevertheless, unrecognized errors in coding or reporting may occur and may be non-random. Lastly, we cannot determine the extent to which spending differences were due to hyperlocal market forces, patient factors, or physician preference. Prior studies suggest that all three factors impact the cost of care.^46^ More study is needed to identify the major drivers for health care spending for individuals with PD. Despite these limitations, our study provides initial evidence that there is substantial geographic variation in health service use and spending for PD. Understanding the drivers of health care costs and needs for individuals with PD is necessary to guide state- and federal-level health policies that support cost efficiency and whole person outcomes for PD patients. Our data are important from a population health and policy perspective, but can also provide meaningful information to clinicians, as knowing the burden of Parkinson’s disease in one’s state is important for physician leaders, and hospital and medical school administrators to plan for and advocate for adequate provider supplies.

## Methods

This study was approved by the Institutional Review Board of the University of Pennsylvania Perelman School of Medicine. A waiver for informed consent was granted.

### Data sources

The data sources for this study were the Medicare Beneficiary Summary File, which contains demographic, geographic, and detailed cost and health care utilization data on every Medicare beneficiary in the US, and Medicare Carrier Files, which contain ICD-9 and procedure codes for diagnoses made by CMS providers (e.g., physicians) in the inpatient and outpatient settings. The study population consisted of individuals aged 65 and above living in the 50 United States and the District of Columbia, who were continuously enrolled in Medicare parts A (which pays for inpatient care) and B (which pays outpatient setting care and provider services) during 2014. We excluded individuals who were enrolled in Health Maintenance Organizations or Medicare Advantage programs, as complete claims and health care use data may not be available for these individuals. We queried the Carrier Files for the ICD-9 codes “332” (Parkinson disease) and “332.0” (paralysis agitans), to identify qualifying Medicare beneficiaries with an active PD diagnosis in the year 2014. Beneficiaries were excluded if they also had diagnostic claims for secondary/drug-induced parkinsonism (“332.1”) or other degenerative disease of the basal ganglia/atypical Parkinson syndromes (“333.0”) since these diseases have a distinct pathophysiology and clinical course.

### State-level PD prevalence

Residence was assigned to one of the 50 states or to the District of Columbia (hereafter referred to simply as “state(s)”) based on the beneficiary mailing address. Crude PD prevalence estimates were calculated by dividing the number of Medicare beneficiaries diagnosed with PD by the total number of Medicare beneficiaries in each state, along with 95% confidence intervals. We also calculated the proportion of PD cases in each state that was (1) aged 85+; (2) female; and (3) dual-eligible. PD is more frequently diagnosed in individuals who are identified as White and male, and PD risk increases with age. Therefore, we also calculated the age-, race-, and sex-adjusted prevalence of PD in each state, using the direct method of standardization and the total Medicare beneficiary population as the standard population.

We have recently used Medicare claims plus data from five other epidemiological studies to produce meta-estimates of the prevalence of PD in North America.^49,50^ These pooled data were used to produce state-level PD prevalence estimates for individuals aged 45 and above, standardized to the 2010 US census population, which we present in comparison to Medicare data-derived prevalence estimates.

For Medicare beneficiaries with and without PD, we extracted data on healthcare utilization (such as the number of emergency room visits, outpatient clinical visits, and inpatient hospitalizations), and the number and refills of covered prescriptions (available for beneficiaries receiving Medicare prescription benefits). Using reimbursement data, we calculated the mean out-of-pocket and CMS cost per individual in each state. State-level rank order lists for PD prevalence (adjusted for age, race, and sex using direct standardization), PD demographic and eligibility characteristics, PD healthcare utilization and costs were produced. Student’s *t*-test with equal variances and Bonferroni correction for multiple comparisons was used to compare direct costs and health service utilization for individuals with and without PD. Choropleth maps for state-level differences in PD population characteristics and cost were produced. Statistical analyses and mapping were performed using Stata/SE version 13.1 (StataCorp LP, College Station, TX, USA).

### Reporting summary

Further information on experimental design is available in the Nature Research Reporting Summary linked to this article.

### Code availability

Analytic code can be made available from the corresponding author upon request.

## Supplementary information

Supplementary Tables 1 and 2

Reporting Summary

## Data Availability

The data that support the findings of this study are available through ResDAC’s CMS Data Request Center and are not publicly available. Due to CMS policy, the authors are unable to provide the data that was used in this study.

## Supplementary information

**Supplementary information** accompanies the paper on the *npj Parkinson’s Disease* website (10.1038/s41531-019-0074-8).
