# Supplementary material for: State-level prevalence, health service use, and spending vary widely among Medicare beneficiaries with Parkinson disease
Source: NPJ Parkinsons Dis. 2019 Jan 24;5:1. doi: 10.1038/s41531-019-0074-8 (PMC6345811; doi:10.1038/s41531-019-0074-8)
Supplement: Supplementary file 1 — Supplementary Tables 1 and 2 [file 41531_2019_74_MOESM1_ESM.pdf]

E-Table 1. Comparison of State-Level Prevalence Estimates			
State	PD Prevalence (per 100, 000)		
	Ages 65+ *	Ages 45+ **	Prevalence estimate ratio (Age 65+/Age 45+)
**Alabama**	1,140.4	577.6	1.97
**Alaska**	935.0	450.3	2.08
**Arizona**	1,173.2	632.1	1.86
**Arkansas**	1,069.4	602.4	1.78
**California**	1,350.7	558.7	2.42
**Colorado**	1,118.7	534.0	2.09
**Connecticut**	1,479.0	578.9	2.55
**Delaware**	1,102.3	596.8	1.85
**District of Columbia**	1,114.8	547.0	2.04
**Florida**	1,469.1	668.5	2.20
**Georgia**	1,054.5	514.0	2.05
**Hawaii**	1,149.3	635.4	1.81
**Idaho**	992.1	593.1	1.67
**Illinois**	1,418.0	566.8	2.50
**Indiana**	1,278.1	571.8	2.24
**Iowa**	1,273.5	627.3	2.03
**Kansas**	1,362.4	602.6	2.26
**Kentucky**	1,122.6	565.5	1.99
**Louisiana**	1,181.4	557.9	2.12
**Maine**	1,061.4	604.6	1.76
**Maryland**	1,227.8	544.8	2.25
**Massachusetts**	1,249.2	577.4	2.16
**Michigan**	1,169.5	581.0	2.01
**Minnesota**	1,195.8	582.2	2.05
**Mississippi**	1,004.9	571.3	1.76
**Missouri**	1,251.1	595.0	2.10
**Montana**	924.8	621.2	1.49
**Nebraska**	1,336.8	608.9	2.20
**Nevada**	1,112.2	557.1	2.00
**New Hampshire**	1,149.7	561.5	2.05
**New Jersey**	1,414.9	566.7	2.50
**New Mexico**	898.8	607.0	1.48
**New York**	1,535.6	579.9	2.65
**North Carolina**	1,018.1	562.5	1.81
**North Dakota**	1,289.8	623.9	2.07
**Ohio**	1,231.1	589.5	2.09
**Oklahoma**	1,052.6	591.8	1.78
**Oregon**	980.7	601.7	1.63
**Pennsylvania**	1,385.6	614.4	2.25
**Rhode Island**	1,255.8	596.8	2.10
**South Carolina**	1,018.2	577.2	1.76
**South Dakota**	1,167.8	626.7	1.86
**Tennessee**	1,245.9	568.3	2.19
**Texas**	1,350.1	526.0	2.57
**Utah**	1,218.8	546.9	2.23
**Vermont**	999.3	590.3	1.69
**Virginia**	1,196.2	548.8	2.18

**Washington**	1,087.2	562.7	1.93
**West Virginia**	1,072.7	615.0	1.74
**Wisconsin**	1,194.2	588.5	2.03
**Wyoming**	814.0	577.8	1.41

***Sample consisting of individuals participating in the Medicare program.**
****Sample consisting of pooled data from the Medicare program, Rochester Epidemiology Project, California Parkinson's Disease Registry, Honolulu-Asia Aging Study, Kaiser Permanente Northern California.**

E-Table 2. Medicare and Individual payments, PD vs. non-PD

Spending Category	*Health Care Use Payment/ Visit Type*	Non-PD (n= 27,145,809)		PD (n=392,214)		p*
		Mean	*Std. Deviation*	*Mean*	*Std. Deviation*	
**Hospital Outpatient**	*Medicare Payments*	1231.57	4475.33	2246.24	5090.56	<0.05
	Beneficiary Payments	334.61	1132.90	588.53	1207.86	<0.05
	Other Payer Payments	21.23	3005.17	22.04	777.91	NS
	Visits	5.49	15.53	12.94	23.05	<0.05
**Acute Inpatient**	*Medicare Payments*	2228.28	9079.98	5369.08	13403.68	<0.05
	Beneficiary Payments	196.66	625.00	484.01	998.97	<0.05
	Other Payer Payments	40.55	2644.52	50.40	2115.92	NS
	Per Diem Payments	67.66	623.27	178.54	975.42	<0.05
	Covered Days	1.03	4.21	2.86	6.96	<0.05
**Other Inpatient**	*Medicare Payments*	305.25	3466.09	1485.60	7590.82	<0.05
	Beneficiary Payments	12.34	421.59	65.21	927.12	<0.05
	Other Payer Payments	1.59	759.72	3.96	603.07	NS
	Per Diem Payments	0.29	35.90	1.44	70.34	<0.05
	Covered Days	0.24	2.67	1.21	6.12	<0.05
**Skilled Nursing Facility**	*Medicare Payments*	748.51	4405.29	3605.40	9763.14	<0.05
	Beneficiary Payments	148.84	1115.03	794.92	2570.48	<0.05
	Other Payer Payments	0.60	125.26	2.31	248.10	<0.05
	Covered Days	1.77	10.39	8.58	23.07	<0.05
**Hospice**	*Medicare Payments*	282.04	3076.97	1195.88	6179.41	<0.05
	Other Payer Payments	0.13	53.62	0.20	50.60	NS
	Covered Days	1.78	19.90	7.51	39.71	<0.05
**Home Health**	*Medicare Payments*	448.88	1992.57	1979.92	4334.88	<0.05
	Other Payer Payments	0.16	63.19	0.47	48.61	NS
	Visits	2.79	15.98	12.44	34.70	<0.05
**Ambulatory Surgery**	*Medicare Payments*	69.17	407.76	85.18	569.95	<0.05
	Beneficiary Payments	17.65	104.83	21.84	146.04	<0.05
	Other Payer Payments	0.97	64.44	0.47	32.78	<0.05
	Events	0.14	0.65	0.17	0.73	<0.05
**Part B Drug**	*Medicare Payments*	311.37	2860.10	341.56	2473.47	<0.05
	Beneficiary Payments	75.65	731.33	83.04	632.35	<0.05
	Other Payer Payments	1.75	234.38	1.28	121.15	<0.05
	Events	2.17	5.86	2.98	5.65	<0.05
**E&M**	*Medicare Payments*	385.99	1195.59	1206.20	2294.08	<0.05
	Beneficiary Payments	109.71	314.43	331.83	598.17	<0.05
	Other Payer Payments	1.46	56.42	2.26	72.53	<0.05
	Events	4.95	15.14	15.80	29.50	<0.05
**Part B Physician**	*Medicare Payments*	42.77	133.33	452.64	1680.09	<0.05
	Beneficiary Payments	11.33	35.20	116.57	431.84	<0.05
	Other Payer Payments	0.85	36.52	0.90	88.80	NS
	Events	0.32	0.95	4.89	16.17	<0.05
**Anesthesia**	*Medicare Payments*	11.50	169.80	64.29	166.95	<0.05
	Beneficiary Payments	3.07	45.41	16.82	43.63	<0.05
	Other Payer Payments	0.03	8.21	0.70	36.25	<0.05
	Events	0.07	1.21	0.48	1.33	<0.05
**Dialysis**	*Medicare Payments*	434.41	1347.26	13.31	186.16	<0.05
	Beneficiary Payments	118.82	348.74	3.50	48.98	<0.05

	Other Payer Payments	3.98	176.96		0.02	6.37	NS
	Events	4.74	12.65		0.09	1.44	<0.05
**Other Procedures**	*Medicare Payments*	139.96	315.88		704.92	1586.76	<0.05
	Beneficiary Payments	37.90	84.75		190.89	410.37	<0.05
	Other Payer Payments	1.31	51.12		3.47	123.65	<0.05
	Events	3.22	5.83		9.35	20.08	<0.05
**Imaging**	*Medicare Payments*	197.36	429.77		225.62	343.96	<0.05
	Beneficiary Payments	24.45	63.29		61.88	92.32	<0.05
	Other Payer Payments	0.79	27.65		1.27	44.22	<0.05
	Events	10.71	18.48		6.21	8.09	<0.05
**Tests**	*Medicare Payments*	114.96	598.24		319.80	509.62	<0.05
	Beneficiary Payments	33.26	162.70		41.02	86.21	<0.05
	Other Payer Payments	0.32	31.54		0.92	30.71	NS
	Events	1.86	5.65		17.02	22.28	<0.05
**DME**	*Medicare Payments*	139.66	1092.81		221.87	833.61	<0.05
	Beneficiary Payments	37.20	281.57		62.63	221.60	<0.05
	Other Payer Payments	0.51	76.79		0.39	33.99	NS
	Events	1.79	10.33		3.34	7.47	<0.05
**Other Part B**	*Medicare Payments*	1231.57	4475.33		452.64	1680.09	<0.05
	Beneficiary Payments	334.61	1132.90		116.57	431.84	<0.05
	Other Payer Payments	21.23	3005.17		0.90	88.80	<0.05
	Events	5.49	15.53		4.89	16.17	<0.05
**Other Health Care Utilization**	*ED Visits w/ Discharge*	0.30	0.90		0.72	1.46	<0.05
	ED Visit w/ Admission	0.16	0.56		0.46	0.94	<0.05
	Acute Inpatient Stays	0.21	0.66		0.56	1.04	<0.05
	Other Inpatient Stays	0.02	0.15		0.08	0.34	<0.05
	Skilled Nursing Facility Stays	0.07	0.34		0.29	0.70	<0.05
	Hospice Days	0.02	0.16		0.09	0.31	<0.05
	Hospital Readmissions	0.03	0.27		0.10	0.44	<0.05
**Part D**	*Total Prescription Costs*	1407.97	5242.06		3235.70	6754.96	<0.05
	Medicare Payments	980.79	4518.86		2303.10	5894.53	<0.05
	Beneficiary Payments	318.94	1217.15		660.93	1590.85	<0.05
	Events	18.26	32.04		42.14	52.05	<0.05
	Fill Count	26.85	40.66		53.50	57.27	<0.05

*Comparison of group means with equal variance assumed, Bonferroni correction for multiple comparisons
Abbreviations: NS= Not statistically significant, ED= Emergency Department
